# Factors Associated with the Introduction of *Mycobacterium avium* spp. Paratuberculosis (MAP) into Dairy Herds in Galicia (North-West Spain): The Perception of Experts

**DOI:** 10.3390/ani11010166

**Published:** 2021-01-12

**Authors:** Francisco Javier Villaamil, Eduardo Yus, Bibiana Benavides, Alberto Allepuz, Sebastián Jesús Moya, Jordi Casal, Carmelo Ortega, Francisco Javier Diéguez

**Affiliations:** 1 ADSG Costa da Morte, 15124 A Coruña, Spain; javieradscm@gmail.com; 2 Institute of Food Analysis and Research, Santiago de Compostela University, 27002 Lugo, Spain; eduardo.yus@usc.es; 3 Animal Health Department, Universidad de Nariño, San Juan de Pasto 52001, Colombia; bibiana.benavides@e-campus.uab.cat; 4 Department of Health and Animal Anatomy, Autonomous University of Barcelona, 08193 Barcelona, Spain; Alberto.Allepuz@uab.cat (A.A.); Sebastian.Moya@uab.cat (S.J.M.); Jordi.Casal@uab.cat (J.C.); 5 Centre de Recerca en Sanitat Animal (CReSA), UAB, IRTA, 08193 Barcelona, Spain; 6 Animal Pathology Department, Veterinary Faculty, Zaragoza University, 50013 Zaragoza, Spain; epidemio@unizar.es; 7 Anatomy, Animal Production and Clinical Veterinary Sciences Department, Veterinary Faculty, Santiago de Compostela University, 27002 Lugo, Spain

**Keywords:** dairy cattle, expert opinion, Johne’s disease, risk analysis, Spain

## Abstract

**Simple Summary:**

Paratuberculosis remains one of the most important diseases of cattle worldwide. Control of disease is difficult and offers important challenges at both diagnostic and management levels. This paper describes a study aimed at quantification of expert opinion on risk factors for paratuberculosis infection in dairy herds in Spain. For this purpose, a panel of nine experts working in the field of paratuberculosis was selected. Risk factors were also included into a questionnaire that was responded to by 93 farms whose sanitary status was known. The most important risk factors for the introduction of MAP, according to expert opinions, were related to purchase and grazing practices. The scores obtained for each farm, based on the expert opinions, allowed MAP positive/MAP negative farms to be discriminated with 68.8% sensitivity and 68.7% specificity. Despite increased awareness of the disease and the fact that several countries are implementing control programs, there is still incomplete understanding of the epidemiology of the disease. This, together with the lack of completely reliable diagnostic methods, makes it of vital importance considering the inter-herd transmission factors in order to prevent the introduction of the disease. Prioritizing the most important factors should be useful for focusing future training initiatives and improving risk-reduction strategies in this economically important industry.

**Abstract:**

This study aimed at quantifying expert opinions on the risk factors involved in *Mycobacterium avium* subsp. *paratuberculosis* (MAP) infection in dairy cattle herds. For this purpose, potential risk factors associated with the introduction of MAP into dairies were chosen based on a literature review and discussions with researchers and veterinarians. For each factor, a decision tree was developed, and key questions were included in each. Answers to these key questions led to different events within each decision tree. An expert opinion workshop was organized (following the recommendations of the OIE), and ordinal values ranging from 0 to 9 (i.e., a null to very high likelihood of infection) were assigned to each event. The potential risk factors were also incorporated into a structured questionnaire that was responded to by 93 farms where the sanitary status against MAP was known. Thereby, based on the values given by the experts and the information collected in the questionnaires, each farm was assigned a score based on their MAP entry risk. From these scores (contrast variable) and using a ROC curve, the cut-off that best discriminated MAP-positive and -negative farms was estimated. The most important risk factors for the introduction of MAP, according to expert opinions, involved purchase and grazing practices related to animals under six months of age. The scores obtained for each farm, also based on the expert opinions, allowed MAP positive/MAP negative farms to be discriminated with 68.8% sensitivity and 68.7% specificity. These data should be useful for focusing future training initiatives and improving risk-reduction strategies in the dairy industry.

## 1. Introduction

*Mycobacterium avium* subspecies *paratuberculosis* (MAP) is the causative agent of paratuberculosis or Johne’s disease (JD), a chronic granulomatous enteric disease that affects domestic and wild ruminants; the disease seems to respond to antibiotics as symptoms weaken but recur after antibiotics are no longer administered. The organism is an acid-fast, Gram-positive and facultative intracellular pathogen that requires iron for growth, dependent on mycobactin, and therefore is incapable of environmental replication [1]. However, MAP is extremely resistant and, in ideal conditions, can survive for up to a year in the environment [2].

This disease causes serious economic losses in dairy farming, mainly as a result of reduced milk yield [3,4], increased susceptibility to other diseases, particularly mammary infections [5], loss of bodyweight [6] and consequential premature culling [7]. Paratuberculosis has also been related to reduced fertility rates [4,8]. In any case, and especially on farms with a low prevalence, the economic impact may be difficult for the farmer to appreciate. MAP is also a long-suspected cause of Crohn’s disease in humans and a recently proposed cause of ulcerative colitis [9].

The primary means of MAP transmission is the fecal–oral route. The bacteria are usually introduced into herds through the purchase of infected but clinically inconspicuous cattle. Introduced animals may be subclinically infected, shed the bacteria and contaminate the environment for several months or even years before clinical signs appear. Other routes, such as the introduction of contaminated feces by vehicles, equipment and visitors; contaminated pasture or water sources; other ruminants; and so on, are less common but may also be involved in the spread of the disease [10,11,12]. Once in the herd, younger animals are the most susceptible to MAP infection, especially shortly after birth, although clinical signs typically do not appear for 3–5 years, reflecting the long incubation period and slow course of the disease. A previous study stated considerable differences in age susceptibility to infection between adults and calves less than six months of age and between adults and calves aged 6–12 months [13].

Bovine paratuberculosis progresses through four main stages, depending on the severity of clinical signs, the potential for shedding organisms into the environment and the ease with which the disease may be detected using current laboratory methods. Stage I is asymptomatic (i.e., subclinical) with the pathogen not detectable in feces. This is followed by a stage (Stage II) in which the disease remains asymptomatic and the pathogen is often shed in insufficient quantity to be detectable by conventional tests. The infected animal may have an altered immune response. In the clinical stage (Stage III), nearly all animals are detectable by PCR or culture of fecal samples and usually have increased antibody detectable by a commercial ELISA test. The animal has gradual weight loss and diarrhea. The appetite remains normal and intermittent diarrhea is often present for weeks. Finally, animals in Stage IV of the disease are weak, emaciated and usually have chronic, profuse diarrhea. Intermandibular edema or bottle jaw is characteristic of this phase of the disease [14].

Control of paratuberculosis has proved to be highly problematic due to the relatively poor performance of diagnostic tests in the initial stages, the prolonged incubation period and extensive environmental survival [10]. The uncertainty surrounding the estimations of paratuberculosis prevalence and its impact also condition control strategies [15].

For this reason, biosecurity measures that reduce transmission opportunities between herds are essential for controlling the disease. However, due to the recommended practices often being laborious, and the difficulties in understanding the negative implications of JD on the part of the farmers, the latter often fail to comply with the recommended practices and end up stopping the control measures. For this reason, reliable information on the main measures for preventing the entry of MAP into herds, as well as an assessment of their relative importance, would be useful for focusing training and awareness programs and improving risk reduction strategies.

The objective of this study was to perform a semi-quantitative risk analysis of factors relating to the entry of MAP into dairy herds, based on expert opinions.

## 2. Material and Methods

### 2.1. Area Description

The study was carried out in Galicia. This region is the main dairy cattle area in Spain, contributing 55% of the farms and 38% of the milk production. The mean Galician herd size per farm is 43 cows, lower than the national average of 59.3, and the farms are still predominantly family owned and managed [16]. In addition, the dairy farming population is characterized by a high density of farms and a low availability of grassland.

Galicia was the first region in Spain to establish a voluntary paratuberculosis control program, in 2004. Blood samples from all farms included in the program were taken annually from cows older than two years for laboratory analysis. The serum was analyzed for anti-MAP antibodies with commercial ELISA (Paratuberculosis Screening Ab; IDEXX, Westbrook, ME, USA) and fecal samples of ELISA-positive samples were analyzed by PCR (ID Gene^®^ Paratuberculosis Duplex, ID.Vet, Grabels, France).

### 2.2. Herds Surveyed and Data Collection

Potential factors associated with the introduction of MAP into dairy herds were selected according to a literature review [15,17,18], as well as discussions with researchers and veterinarians at Santiago de Compostela University and the Autonomous University of Barcelona, regional government veterinarians and cattle veterinarians from diverse subspecialties. The considered factors were included in a structured questionnaire that was given to 93 dairy farms from Galicia [19]. The questionnaires were completed, between July 2017 and April 2018, through personal interviews with the farmer and the veterinarian responsible for the control program at each farm (the 93 farms formed part of the voluntary MAP control program).

The questionnaire comprised closed questions and included three main sections: (I) animal movements (e.g., frequency of introductions and sanitary status in the farm of origin, cattle fairs or competitions, pastures and small ruminants on that same farm); (II) movements and types of vehicles and equipment (for live and dead animal transport, manure, slurry and feeding vehicles, machinery or materials) and biosecurity-related measures (e.g., vehicles may enter the farm perimeter and vehicles may enter with other animals); and (III) visitors and staff (e.g., external workers; frequency of visits by professionals, such as veterinarians and technicians, or non-professionals, such as neighboring farmers, who come into contact with the animals; and use of protective clothing). The questionnaire gathered information on the farming practices over the last two years, including the numbers of both animal purchases and external employees.

### 2.3. Expert Group Workshop

**Figure 1:**
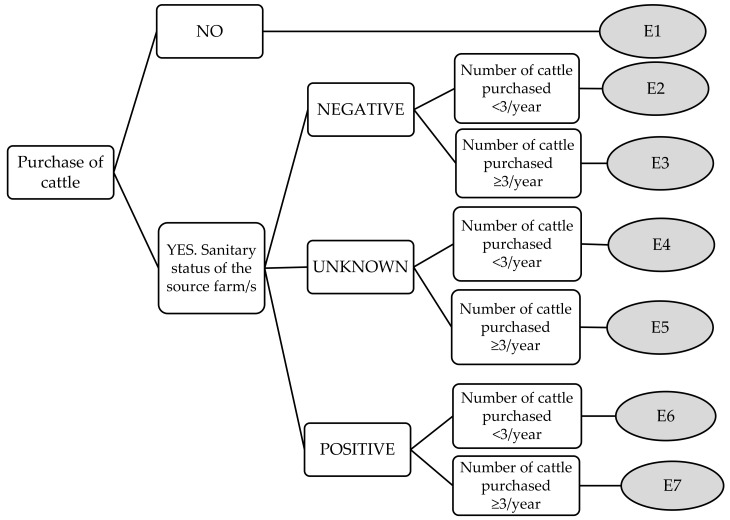
Purchase of cattle decision tree.

**Figure A1:**
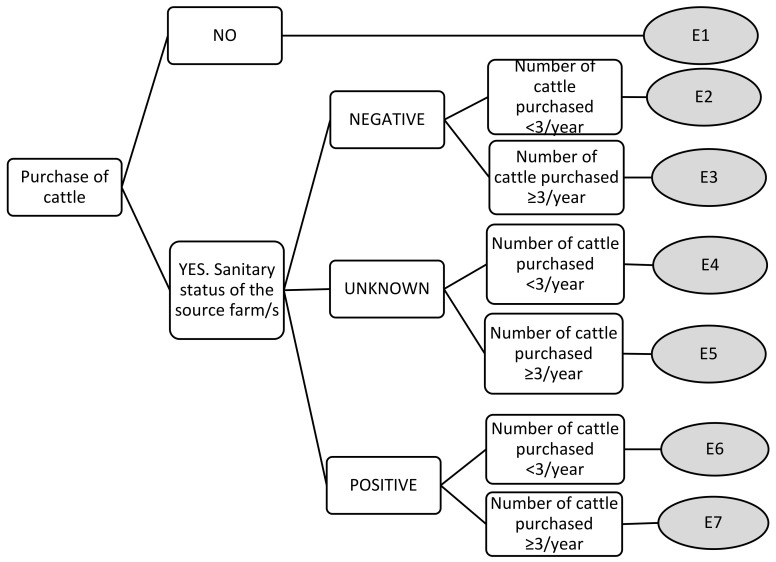

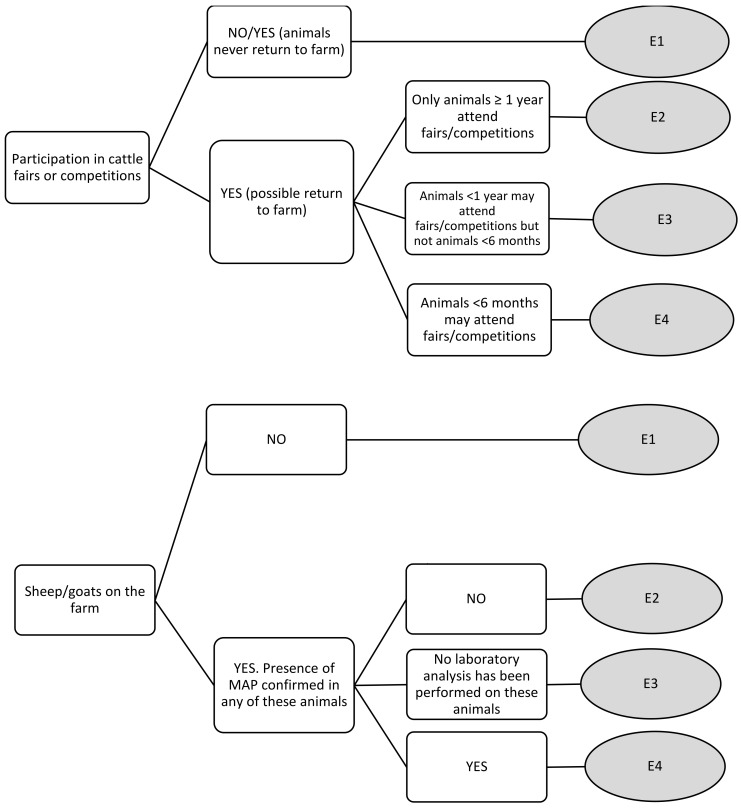

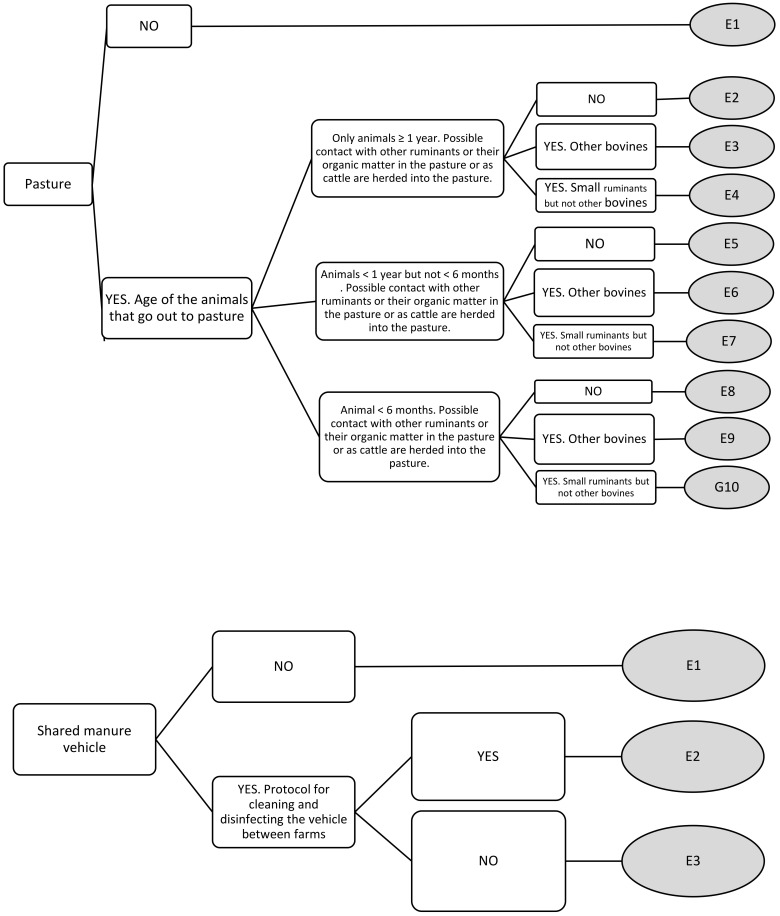

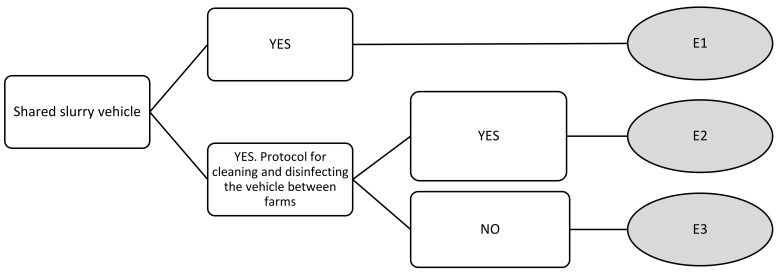

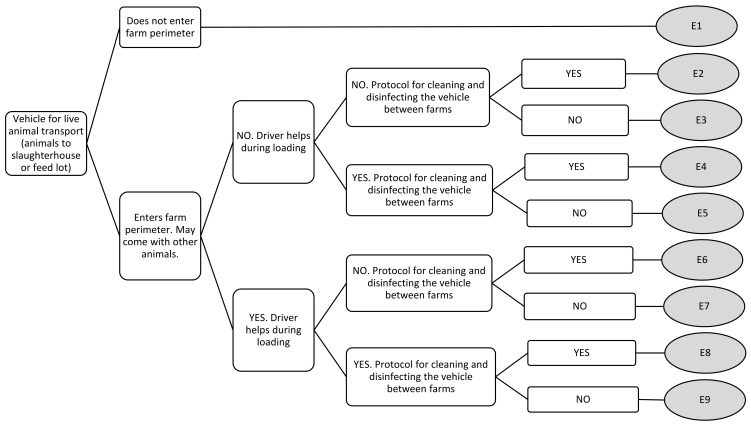

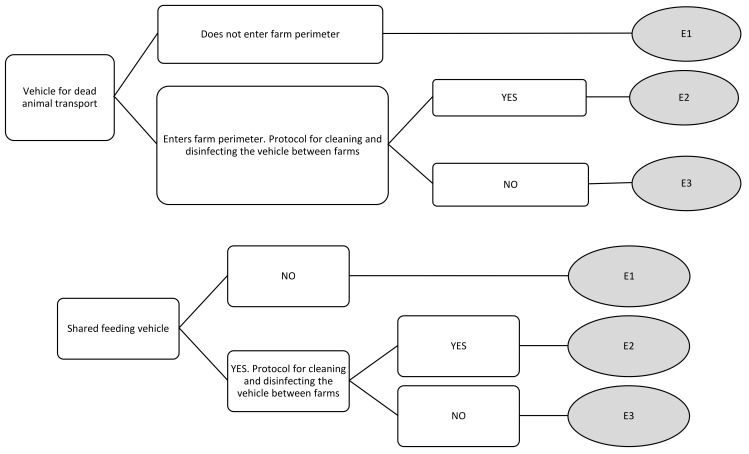

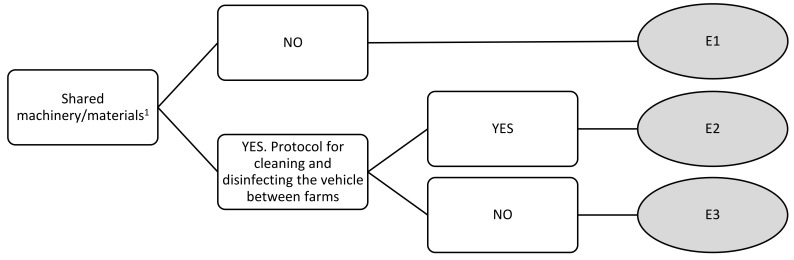

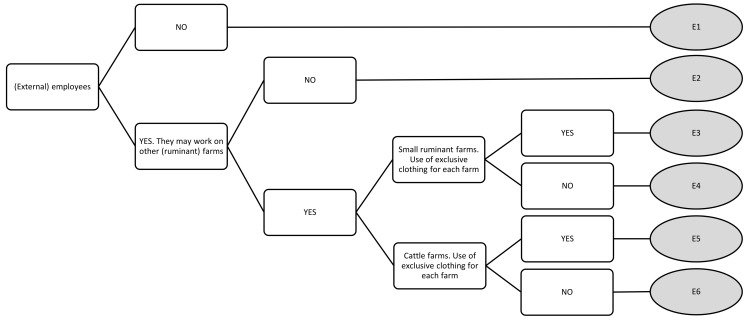

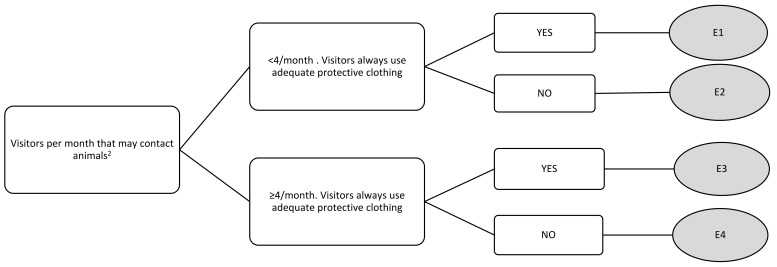
Decision tree diagrams. The sanitary status of the origin herd was considered known if it had a testing protocol comparable or superior to that in the herds in the control program in Galicia (negative when all animals tested negative in all tests performed in the last sampling campaign prior to the purchase of the animals or positive if at least one animal tested positive in any test). The sanitary status of the origin herd was considered unknown in any other case (either no testing protocols or testing protocols inferior to those implemented in Galicia). The median frequency of entries in those farms that purchase cattle in the study population is three. ^1^ Tractors, wagons, cistern, slurry whisk, baler or calving, cleaning, or harvesting materials. ^2^ The median number of visitors in the study population is four. Adequate protective clothing was defined as clean coveralls or overcoats and boots that are provided by the farmer before entering the cattle area.

Moreover, for each factor considered, a decision tree was developed, and key questions were included in each. Answers to these key questions led to different events within each decision tree. Figure 1 shows the decision tree for the purchase of animals. The other decision trees are included in Figure A1 (Appendix A). For example, event E3 in Figure 1 corresponds to a herd for which cattle had been purchased over the preceding two years, the sanitary status of the origin farm/s was negative and the mean number of purchased cattle was ≥3/year.

Subsequently, an expert opinion workshop was organized to gather opinions on the risk that each event from the decision trees would pose for the possible entry of MAP into dairy herds. This was implemented following the recommendations of the Handbook on Import Risk Analysis for Animals and Animal Products [20]:

- Initially, nine vets from different subspecialties (laboratory personnel in charge of processing MAP control program samples, practitioners, veterinarians in charge of MAP control programs, researchers specializing in epidemiology and infectious diseases in cattle, cattle association veterinarians and veterinarians from pharmaceutical laboratories) were selected, based on their experience, proximity and knowledge of the disease. Information on the background of the participants is provided as Table A1 (Appendix B).
- Next, the decision trees were sent to each veterinarian, together with an appropriate explanation of the method, where each event was to be assigned a score (on an ordinal scale from 0 to 9) based on the risk that, in the opinion of each expert, this event could pose for the entry of the disease into a dairy herd [21].
- Finally, a workshop for experts was held on December 2019 in the Veterinary Faculty of Lugo (Spain). During the workshop, doubts and possible misunderstandings were discussed and resolved. In addition, histograms showing the scores assigned by the participants were presented. These histograms were discussed with the entire group, and the participants had the chance to change their assigned scores if they considered that they had over- or underestimated any of the events.

### 2.4. Data Analysis

Initially, the mean score (along with median, maximum and minimum scores) assigned by the participating experts to each event from the decision trees was calculated. From these means, and using the data collected from the 93 questionnaires, the score that each farm would obtain for each decision tree was recorded. For example, in the case of a farm that had purchased ≥3 animals/year from negative herds (Event 3 in the decision tree for the purchase of animals, Figure 1), the mean score that experts assigned to that event would be recorded for this farm. Finally, by adding up all the scores obtained by each farm for all of the decision trees, a total score was assigned to each farm.

The 93 herds included in the study were classified as either MAP positive or MAP negative based on the laboratory results obtained in the two annual sampling campaigns performed prior to the completion of the questionnaire. Herds with no seropositive cows in either annual sampling were classified as negative (*n* = 67). Herds with the presence of the bacterium confirmed by either PCR or seropositivity ≥15% without this being confirmed in either of the annual sampling campaigns were classified as positive (*n* = 16). To minimize misclassification, the remaining herds, which were seropositive ≤15% without confirmation of the bacterium or seronegative herds with only one annual sampling campaign (as they had recently joined the program) (*n* = 10), were not included in this part of the analysis.

Total scores in positive and negative farms were compared using ANOVA. Additionally, from the total scores (contrast variable) and using a ROC curve, the cut-off that best discriminated positive and negative farms was estimated.

Additionally, for each positive farm, the two highest scoring events recorded on that farm, according to expert opinion, were also provided.

## 3. Results

The mean, median, maximum and minimum scores given by veterinarians for each event in each decision tree are presented in Table 1.

According to the opinions of experts, the events with the highest assigned scores were: purchase ≥ 3 animals/year from positive farms (mean score 8.89 out of 9); purchase < 3 animals/year from positive farms (8.11/9); MAP-positive small ruminants on the farm (8/9); animals < 6 months pastured with possible contact with cattle from other farms (7.67/9); purchase ≥ 3 animals/year from farm(s) with unknown sanitary status (7.33/9); participation in fairs/contests with return of the animals (7/9); and animals < 6 months pastured with possible contact with small ruminants from other farms (7/9) (Table 1).

The maximum total score that a farm could obtain, if all the decision trees had the highest scoring event, was 77.90. The mean total score (by adding up the scores obtained for all of the decision trees) was 23.26 (S.D. = 5.92) for negative farms and 30.61 (S.D. = 10.72) for positive farms (*p* < 0.001). According to the ROC curve, the cut-off score that best discriminated positive and negative farms was 25.94. This cut-off provides 68.8% (46/67) sensitivity and 68.7% (11/16) specificity (Table 2). Five out of 51 farms (9.8%) with scores below 25.94 were positive, while this percentage increased to 34.4% (11/32) among those farms with scores equal to or higher than 25.94 (Table 2). Additionally, all farms with a total score higher than 42.99 (*n* = 3) were positive.

For each positive herd, the two highest scoring events according to the expert group are presented in Table 3. In 5 out of 16 positive farms (31.2%), the highest scoring event was related to the purchase of cattle. Interestingly, in 6 out of 16 (37.5%), it was related to the entry of various vehicles with no implementation of cleaning and disinfection protocols.

## 4. Discussion

In Spain, official MAP control programs in cattle are only carried out in the Galicia region, which, as mentioned, is the main dairy cattle area of the country. These programs are voluntary and conducted through farmer associations, called Livestock Health Defense Associations (known as ADSG in Spanish). They are coordinated by the regional government. Nowadays, ADSGs include 55.2% of the herds and 65.0% of Galician bovine census. The regional government covers the cost of laboratory tests and part of the salary of the veterinarian responsible [22]. Control efforts and programs against MAP in cattle are very diverse among European states (although paratuberculosis is notifiable in most of them). As in Galicia, the majority of countries have voluntary control programs, also coordinated at regional level in many cases. In line, government funding is frequently involved, but operations tend to be supported by farmers and their organizations and not by government alone [23,24]

The present paper focuses on factors related to bioexclusion or external biosecurity, that is, factors associated with the entry of MAP into a herd. In this way, it differs from many other previous studies where bioexclusion was assessed together with biocontainment measures. The results of the expert workshop emphasize the relative importance of the considered biosecurity measures, with animal movement (i.e., the frequency of introductions and sanitary status in the farms of origin, pasture, small ruminants on the same farm) playing a central role. However, other biosecurity measures should not be underestimated, particularly the control of shared vehicles/materials and visitors that have contact with the animals.

Most previous studies based on regression analysis (in other populations and using other diagnostic approaches) coincided in pointing out the importance of animal purchases, indicating odds ratios ranging from 1.31 to 5.44 [11,12,18,25]. In contrast, McAllon et al. (2017) found that purchasing behavior over the preceding 10 years was not significant [10].

In Galicia, 31% of the farms purchased cattle [26]. The primary difference between this study and previous ones is that, in Galicia, farms that are part of the control program are required to test all newly purchased animals using antibody ELISA test. However, given the limitations of the serum ELISA test at the individual level [27], it seems necessary to have comprehensive information on the overall status of the farm of origin [11]. Purchase of cattle is the highest scoring event in 5 out of the 16 positive farms in the study. Although for farms in the control program it is compulsory to carry out an antibody ELISA test on purchased animals, there are no obligations to implement other biosecurity measures [28].

Although purchased cattle are considered to be the main risk factor for disease entry to dairy farms [11,12,18,25], the importance of other measures should not be underestimated and may result in less use of monetary and labor resources intended for control programs. Other practices to which veterinarians give high scores (>7 out of 9) are less frequently observed in the study population [19]. Although it has been suggested that interspecies transmission of some MAP strains may not be a rare event if there is close contact between different species at the farm level [29], a small proportion of farms kept small ruminants. Furthermore, animal movement to cattle fairs or competitions (with return of the animals) is a possible disease transmission pathway for few farms in the region, and farms that allow calves to graze are also scarce [19]. In any case, within a herd, it is the younger animals that are the most susceptible to contracting MAP, and this tends to move slowly through soils and remain on the grass and in the upper layers of pasture soil, posing an infection hazard for grazing animals [30]. The presence of small ruminants on the farm is the highest scoring event in 1 of the 16 positive farms in the study, whereas the grazing of animals under six months of age is the highest scoring event for two positive farms.

However, other factors that are observed more frequently on farms in Galicia and which were scored as >6 out of 9, according to expert opinion, should also be considered, particularly shared vehicles that enter the farm perimeter with no cleaning and disinfection implemented between farms (i.e., manure, slurry or live animal transport vehicles) or visitors who do not wear protective clothing. These factors have been less well evaluated in previous studies but, according to the scores given by the experts, they could be of great importance as they are events that occur frequently in the study population [19]. Shared vehicles or visitors with no protective clothing are the highest scoring event in 6 out of 16 positive farms.

Certain environments on the farm were more likely to be contaminated with MAP and had higher average contamination levels. These high-risk areas included manure storage areas and shared alleyways, in other words parts of the farm where the manure of adult cows is mixed [31]. Manure vehicles are the vehicles to which the experts assigned the highest risk. Additionally, visitor footwear has previously been considered a high-risk fomite for the dispersion of dust-related MAP outside the barn [32].

Given the characteristic of JD, the main control point to help avoid the infection of the farms should be the implementation of robust biosecurity measures that prevent the introduction of the bacteria to the farm. Based on the opinions of the participating experts, it was possible to assign a score to each farm based on its level of biosecurity, which discriminated farms with a high and low risk of infection, with more than 68% sensitivity and specificity. The results suggest that control programs could identify and emphasize the practices that pose the highest risk for each farm.

A possible limitation of the method is that PCR tests were only performed on seropositive animals. There is the possibility that the cows are ELISA-negative but fecal culture positive; this could be the result of passive shedding due to the through-pass of MAP in the intestinal tract [33]. However, it has been indicated that ELISA-positive cows were up to 8.8 times more likely to shed the bacteria [34]. In addition, for the study, the laboratory data were assessed at the herd, not individual, level.

## 5. Conclusions

Given the importance of risk factor management in the control of MAP, conducting risk factor studies in the regions where control programs are to be implemented is highly advisable. Despite the inherent limitations of this study, we believe it provides a comprehensive overview of the main factors related to the entry of MAP into dairy farms. These data should be useful for focusing future training initiatives and improving risk-reduction strategies in this economically important industry.

## Figures and Tables

**Table 1:** Mean ordinal values for each event together with the, minimum, median and maximum values according to expert opinion.

		Mean	Median	Maximum	Minimum
Purchase of cattle	E1	0	0	0	0
	E2	2	2	3	1
	E3	3.11	3	4	2
	E4	6.22	6	8	5
	E5	7.33	7	9	6
	E6	8.11	8	9	7
	E7	8.89	9	9	8
Participation in cattle fairs or competitions	E1	0	0	0	0
	E2	3.44	3	5	1
	E3	5.22	5	7	4
	E4	7	7	9	5
Sheep/goats on the farm	E1	0	0	0	0
	E2	2.22	2	3	0
	E3	5.44	5	7	4
	E4	8	9	9	6
Pasturing	E1	0	0	0	0
	E2	1.44	1	3	0
	E3	4.44	5	5	3
	E4	3.78	4	5	2
	E5	1.33	1	3	0
	E6	6.22	6	7	5
	E7	5.55	6	7	3
	E8	2.22	2	5	0
	E9	7.67	7	9	6
	E10	7	7	9	4
Shared manure vehicle	E1	0	0	0	0
	E2	3	3	5	1
	E3	6.89	7	9	4
Shared slurry vehicle	E1	0	0	0	0
	E2	2.89	2	5	1
	E3	6.67	7	9	4
Vehicle for live animal transport (animals to slaughterhouse or feed lot)	E1	0	0	0	0
	E2	1.11	1	2	0
	E3	2.67	2	5	1
	E4	2.11	2	4	1
	E5	3.44	3	5	2
	E6	3.22	3	5	2
	E7	4.78	5	6	3
	E8	4.44	4	7	2
	E9	6.22	7	8	3
Vehicle for dead animal transport	E1	0	0	0	0
	E2	2.11	2	4	1
	E3	4.55	5	6	3
Shared feed vehicle	E1	0	0	0	0
	E2	2.55	2	4	2
	E3	4.78	5	6	3
Shared machinery/materials	E1	0	0	0	0
	E2	2.67	3	4	2
	E3	5.33	5	7	4
(External) employees	E1	0	0	0	0
	E2	0.55	1	1	0
	E3	1.78	2	3	0
	E4	5.22	5	7	3
	E5	2.22	3	4	0
	E6	5.78	6	7	3
Visitors per month that could contact animals	E1	2.11	2	3	0
	E2	4.55	5	5	4
	E3	2.78	3	5	0
	E4	6.11	7	7	4

**Table 2:** Cross-classification of the MAP status of the farms (obtained from the laboratory tests performed as part of the control program) and the total score for the farms (obtained from the farm management practices according to the opinions of a group of experts).

		Farm MAP Status		Total
		Negative	Positive	
Total score	<25.94	46	5	51
	≥25.94	21	11	32
Total		67	16	83

**Table 3:** Highest two scoring events according to the mean values assigned by the expert group in the 16 infected farms in Galicia (mean score assigned to this event).

Farm	Highest Scoring Event	Second-Highest Scoring Event
1	Shared manure vehicle and no cleaning and disinfection protocol between farms (6.89)	Shared slurry vehicle and no cleaning and disinfection protocol between farms (6.67)
2	Animals < 6 months go to pasture with possible contact with cattle from other farms (7.67)	Purchase ≥ 3 animals/year from farm with unknown sanitary status (7.33)
3	Purchase ≥ 3 animals/year from farm with unknown sanitary status (7.33)	Shared manure vehicle and no cleaning and disinfection protocol between farms (6.89)
4	Shared manure vehicle and no cleaning and disinfection protocol between farms (6.89)	Visitor/month > 4 without protective clothing (6.11)
5	Purchase ≥ 3 animals/year from farm with unknown sanitary status (7.33)	Shared manure vehicle and no cleaning and disinfection protocol between farms (6.89)
6	Purchase ≥ 3 animals/year from farm with unknown sanitary status (7.33)	Vehicle for live animal transport enters farm perimeter, with other animals, driver helps with loading and there are no cleaning and disinfection protocols between farms (6.22)
7	Vehicle for live animal transport enters farm perimeter, with other animals, driver helps with loading and there are no cleaning and disinfection protocols between farms (6.22)	Shared feeding vehicle and no cleaning and disinfection protocol between farms (4.78)
8	Small ruminants in the farm with unknown sanitary status (5.44)	Vehicle for live animal transport enters farm perimeter, with other animals, driver does not help with loading and there are no cleaning and disinfection protocols between farms (4.78)
9	Vehicle for live animal transport enters farm perimeter, with other animals, driver helps with loading and there are no cleaning and disinfection protocols between farms (6.22)	Visitors/month > 4 without protective clothing (6.11)
10	Vehicle for live animal transport enters farm perimeter, with other animals, driver helps with loading and there are no cleaning and disinfection protocols between farms (6.22))	Visitors/month > 4 without protective clothing (6.11)
11	Purchase ≥ 3 animals/year from farm with unknown sanitary status (7.33)	Shared feeding vehicle and no cleaning and disinfection protocol between farms (4.78)
12	Purchase ≥ 3 animals/year from farm with unknown sanitary status (7.33)	Shared slurry vehicle and no cleaning and disinfection protocol between farms (6.67)
13	Visitors/month > 4 without protective clothing (6.11)	Shared feeding vehicle and no cleaning and disinfection protocol between farms (4.78)
14	Animals < 6 months go to pasture with possible contact with cattle from other farms (7.67)	Visitors/month > 4 without protective clothing (6.11)
15	Visitors/month > 4 without protective clothing (6.11)	Vehicle for live animal transport enters farm perimeter, with other animals, driver does not help with loading and there are no cleaning and disinfection protocols between farms (4.78)
16	Shared feeding vehicle and no cleaning and disinfection protocol between farms (4.78)	Visitors/month < 4 without protective clothing (4.55)

## Data Availability

The data presented in this study are available on request from the corresponding author.

## Appendix B

**Table A1:** Background and expertise of the different experts who participated in the workshop.

	Position. Institution	Expertise
1	Head of the molecular biology service. Animal health and production laboratory (Xunta de Galicia).	Around 28 years working on serology and molecular biology of infectious cattle diseases.
2	Chief vet. Sanitary Defense Group of Galicia	Responsible for the MAP control program in 4 municipalities in Galicia over the past 12 years. Previously 15 years of experience as free cattle practitioner.
3	Chief vet. Sanitary Defense Group of Galicia	Responsible for the MAP control program in 5 municipalities in Galicia over the past 15 years.
4	Professor and researcher. Animal Health. Veterinary Faculty. Zaragoza University	Around 30 years working on infectious ruminant diseases, mainly with risk analysis and transmission models.
5	Professor and researcher. Animal Health. Veterinary Faculty. Autonomous University of Barcelona	Around 20 years working on infectious cattle diseases mainly with risk and spatial analysis.
6	Professor and researcher. Animal Health. Veterinary Faculty. Santiago de Compostela University	Around 35 years working on infectious cattle diseases mainly with diagnostic techniques and transmission models.
7	Head of Dairy Herd Improvement Program, Galicia	Around 33 years of experience with dairy cattle. Has worked as a practitioner, nutritionist, head of a farming cooperative and during the last 23 years as head of the Dairy Herd Improvement Program in Galicia.
8	Cattle field technician. Pharmaceutical laboratory	Around 19 years of experience with dairy cattle. Free practitioner and has spent the last 5 years as a dairy cattle field technician at a pharmaceutical laboratory.
9	Free cattle practitioner	Around 9 years of experience as a free dairy cattle practitioner.
